# Defining Valid Activity Monitor Data: A Multimethod Analysis of Weight-Loss Intervention Participants’ Barriers to Wear and First 100 Days of Physical Activity

**DOI:** 10.3390/informatics8020039

**Published:** 2021-06-06

**Authors:** Stephanie L. Orstad, Lauren Gerchow, Nikhil R. Patel, Meghana Reddy, Christina Hernandez, Dawn K. Wilson, Melanie Jay

**Affiliations:** 1 Department of Medicine, New York University Grossman School of Medicine, New York, NY 10016, USA;; 2 Rory Meyers College of Nursing, New York University, New York, NY 10010, USA;; 3 Medical School, University of Texas Southwestern Medical Center, Dallas, TX 75390, USA;; 4 Department of Psychology, University of South Carolina, Columbia, SC 29208, USA;; 5 Veterans Affairs, New York Harbor Healthcare System, New York, NY 10010, USA

**Keywords:** wearables, fitness tracker, accelerometer, pedometer, self-monitoring, exercise, steps, obesity, Hispanic, Latina, mHealth

## Abstract

Despite the popularity of commercially available wearable activity monitors (WAMs), there is a paucity of consistent methodology for analyzing large amounts of accelerometer data from these devices. This multimethod study aimed to inform appropriate Fitbit wear thresholds for physical activity (PA) outcomes assessment in a sample of 616 low-income, majority Latina patients with obesity enrolled in a behavioral weight-loss intervention. Secondly, this study aimed to understand intervention participants’ barriers to Fitbit use. We applied a heart rate (HR) criterion (≥10 h/day) and a step count (SC) criterion (≥1000 steps/day) to 100 days of continuous activity monitor data. We examined the prevalence of valid wear and PA outcomes between analytic subgroups of participants who met the HR criterion, SC criterion, or both. We undertook qualitative analysis of research staff notes and participant interviews to explore barriers to valid Fitbit data collection. Overall, one in three participants did not meet the SC criterion for valid wear in Weeks 1 and 13; however, we found the SC criterion to be more inclusive of participants who did not use a smartphone than the HR criterion. Older age, higher body mass index (BMI), barriers to smartphone use, device storage issues, and negative emotional responses to WAM-based self-monitoring may predict higher proportions of invalid WAM data in weight-loss intervention research.

## 1. Introduction

Wearable activity monitors (WAMs), such as the Fitbit^®^, are widely available and highly acceptable behavior change tools [1,2]. WAMs capture continuous physical activity (PA) behavior in real time and provide immediate feedback to the user on goal progress. Meta-analyses confirm that activity monitors delivered with behavioral instruction, goal setting, and regular feedback are effective interventions for PA promotion and weight and diabetes management in adults with obesity [3–7]. WAMs are commonly deployed in PA research not only to change behavior but also to assess PA, given the precision and objectivity of their accelerometer function when compared to self-reported PA measures [8]. For instance, Fitbit has been used in over 1000 research projects since 2012 [9] and has been registered in ClinicalTrials.gov 10 times more frequently than other WAM brands [10,11]. Fitbit devices are considered among the most accurate commercially available WAMs [11]. Fitbit validity results for free-living moderate-vigorous physical activity (MVPA) range from moderate to strong correlation with research-grade accelerometers (Spearman’s *r* = 0.56 [12], 0.86 [13], and 0.88–0.91 [14]). Despite the benefits of objective data, continuous measurement of PA over time results in large quantities of data to process. Determining an appropriate analytic approach requires skills in PA assessment and data analysis software [15], but also benefits from interdisciplinary decision making and inquiry into participants’ lived experience.

One such decision researchers must make when analyzing accelerometer data is how to define sufficient WAM “wear adherence” [16,17]. Wear is a measurement of the quantity of time that a participant correctly positions an adequately charged and functioning device on one’s person, often verified via PA or device-specific data such as heart rate, activity counts or intensities, steps and/or sync status. While protocols for calculating wear versus non-wear time for research-grade accelerometers such as the ActiGraph^™^ are generally well established [18,19], no such consensus in protocol exists for commercially available WAMs used in PA intervention research [20]. Interventions reporting PA outcomes measured using commercially available WAMs too frequently fail to describe measurement protocols in detail. In a review of accelerometer protocols for behavioral interventions [21], nearly half (44.2%) did not report the minimum number of days or hours of wear required to include a participant’s data in the analysis. In addition, only eight (17.0%) applied data inclusion criteria of ≥10 h of wear time on at least four out of seven continuous days (standards similar to those applied in PA surveillance [19,22]). The paucity of thorough protocol descriptions for even the most frequently deployed or well-accepted devices impedes design replicability and thus, the ability to make informed decisions about the appropriateness of such devices for capturing PA outcomes in various user groups [20,21,23].

Among intervention studies that describe assessment protocols, there is inconsistency in the type of data and criteria used to assess a valid day of WAM wear. For instance, some studies have applied a daily step-count minimum, which ranged from 300 to 2000 steps per day [24–28]. Other studies utilizing heart rate data have used various definitions of valid wear, including >10 h/day [29], ≥600 1-min episodes of nonzero HR counts [30], and <10% of day with missing HR readings [31]. The minimum number of days considered to gain an accurate picture of an individual’s habitual activity usually ranged from three to seven per week. There were also inconsistencies or omissions in whether periods of interruption in the data were allowed. Inconsistency in definitions of wear for commercially available WAMs suggests that the controllable and uncontrollable barriers to achieving sufficient wear to approximate PA levels at various assessment time points also may not be well defined. Even if participants achieve high compliance to wear protocols established for PA surveillance, difficulties with consumer-grade device functionality, data storage limits, and access to smartphone technology threaten the completeness and validity of the data.

Despite their popularity, decay in sustained use of and engagement with WAMs is expected over time [25,32,33]. A systematic review and content analysis of barriers to and facilitators of engagement with remote measurement technology revealed that, while lack of motivation and worsened health status contributed, the most prominent reasons for disengagement were related to technological difficulties and malfunction [34], which often are beyond the user’s control. WAM acceptability and usability studies have also found that adequate instruction on WAM setup and ease of use are influential factors in sustained WAM wear [35–39], and that more attention should be given to technical aspects and user experience [32]. Associations between demographic and socioeconomic factors and adherence to app-based PA interventions are mixed, though older participants tend to be more adherent [40]. WAM acceptability and barriers to wear are understudied phenomena in socioeconomically and racially/ethnically diverse populations who are disproportionately affected by obesity and its related comorbidities [41]. Thus, there is a critical need to understand barriers to WAM wear and collection of valid data in order to improve PA assessment and promotion in diverse samples of adults with obesity [5,42].

The overarching objectives of this research were twofold. The first was to inform appropriate WAM wear thresholds for low-income, majority Latina patients with obesity enrolled in a behavioral weight-loss intervention. The second was to understand participants’ barriers to using a Fitbit activity monitor during the intervention. These aims can inform WAM protocol decisions for measuring change in PA outcomes in future intervention studies. To address the first objective, we examined continuous Fitbit activity monitor data during participants’ first 100 days in the weight-loss intervention and applied two different definitions of wear to (1a) describe the proportion of participants that met each wear criterion, (1b) examine differences between groups meeting vs. not meeting each wear criterion, and (1c) compare and test concordance between mean daily steps and MVPA outcomes derived from each wear criterion. To address the second objective, we analyzed responses from baseline, 1-, 2-, and 3-month study visit notes, 6-month exit interviews and post-study (12+ month) in-depth interviews to (2a) categorize issues documented by research staff that likely accounted for participants not meeting each wear criterion, (2b) categorize participants’ dislikes with regard to wearing the Fitbit activity monitor, and (2c) identify additional themes that may account for participants not meeting wear criteria. We discuss PA measurement considerations, device acceptability and recommendations for using WAMs with comparable populations, and avenues for future research.

## 2. Materials and Methods

### 2.1. Participants

The Financial Incentives foR Weight Reduction (FIReWoRk) study was a randomized controlled trial to compare the effectiveness of three approaches to weight loss among primary care patients with obesity. The primary outcome was the percentage of patients who achieve a ≥5% reduction in baseline weight at six months. The FIReWoRk protocol has been described in detail previously [43]. Briefly, adults aged 18 to 70 years with a body mass index (BMI) ≥ 30 kg/m^2^ were recruited from three primary care clinics serving racially/ethnically diverse, medically underserved populations in which the prevalence of obesity is above the national average. The clinics were part of “safety net” medical centers in New York City (NYC) and Los Angeles (LA) that provide healthcare for individuals regardless of their insurance status or ability to pay. To be eligible for the study, patients had to reside in a census tract associated with the lowest 40% of 2015 median household income in the NYC/Tri-State and LA County areas (approximately < USD 40,000 per year) [44]. Patients with obesity identified as potentially eligible via queries of electronic health record (EHR) systems received study announcements by mail and follow-up phone calls to invite them to participate. Primary care providers and pamphlets in clinic waiting areas also referred patients to the study. Ultimately, about 5% of patients yielded in the EHR query were eligible and enrolled in the FIReWoRk study (81% of participants were female, 73% identified as Hispanic, and 69% spoke Spanish).

### 2.2. Intervention

All participants received a 1-year commercial weight-loss program membership, self-monitoring tools (bathroom scale, food journal and Fitbit), health education and monthly check-in visits with an interventionist. In addition to these resources, those in the two financial incentives intervention groups could earn up to USD 750 over six months for: (1) participating in an intensive weight management program, self-monitoring weight and diet, and increasing MVPA (goal-directed arm); or (2) achieving a ≥1.5% to ≥5% reduction in their baseline weight (outcome-based arm).

The interventionist communicated that the PA goal was to accumulate ≥75 aerobic activity minutes per week (which increased to ≥150 min per week after three months to approximate PA guidelines). They instructed the participant to wear a commercially available activity monitor (Fitbit Alta HR^™^ or Fitbit Inspire HR^™^) on their non-dominant wrist at all times, except during bathing and swimming. Participants were encouraged, but not required, to wear the device during sleep. The interventionist demonstrated how to charge the device and sync it via Bluetooth with a smartphone, thereby allowing PA data to be recorded. They also assisted the participant in setting up their Fitbit app and online account so that they could access the features available through their smartphone or computer. At a 3-month study visit, the interventionist checked that the device had been syncing, troubleshot issues, and verified whether the participant met their PA goal during each week of the previous month by viewing activity data collected in the participant’s Fitbit account. Participants were not incentivized for Fitbit wear. Goal-directed participants received incentives for MVPA goal attainment. All participants were compensated for each study visit they completed.

### 2.3. Activity Monitor and Data Collection

The Alta HR and Inspire HR include accelerometer, pedometer, and heart rate monitor functions. Proprietary Fitbit algorithms take into account the device’s accelerometer movement and heart rate data, applying minute-by-minute metabolic equivalents to estimate activity intensity. In 2015, Fitbit improved their algorithm to more closely align with 2008 PA guidelines for adults [45] so that their definition of time spent in ‘moderate/fairly active’ and/or ‘intense/very active’ minutes must occur in bouts of ≥10 continuous minutes for a minute to be classified as an ‘active minute’. Though 2018 PA guidelines recognize the health benefits of any time spent in PA, we considered ≥150 Fitbit active minutes per week an approximation of the recommended ≥150 weekly minutes of moderate-vigorous physical activity (MVPA) [46]. Sensor data are stored in the device for variable lengths of time based on the memory required. For example, simple daily step count totals are stored for up to 30 days, whereas daily active minute totals, which depend on minute-by-minute heart rate readings, are stored for seven days before deletion. Users can upload data stored on the device to Fitabase^™^ at any given time via a syncing feature. Fitabase is an independent affiliate of Fitbit that allows researchers to centrally access data from multiple Fitbit wearable devices (Small Steps Labs LLC, San Diego, CA, USA). We downloaded the data for this study from Fitabase on September 17, 2020. Since participants’ active minutes (i.e., FairlyActiveMinutes + VeryActiveMinutes), heart rate, and step count data were required for the analysis, we generated a Fitabase report with metrics including participant-level Intensity (day totals), Heart Rate (1-min), and Steps (day totals). Step totals were also available in 1-min intervals, but we opted to use day totals since they were less vulnerable to deletion due to device storage limits.

### 2.4. Definitions of Valid Activity Monitor Wear

Heart rate (HR) criterion. We defined two criteria for independently determining whether a participant wore the activity monitor for a sufficient length of time in any given day to reflect the whole of their activity for the day, heretofore referred to as a valid wear day. Since 24 h per day of continuous data were possible, we considered a day to begin and end at midnight. This first criterion, the “heart rate” (HR) criterion, is based on daily wear time established for measuring PA minutes per week with research-grade accelerometers (i.e., ≥10 h) [18,19]. We used the Fitbit minute-by-minute heart rate output (i.e., beats per minute) to define a valid wear day as any day with ≥10 h of continuous heart rate recordings, allowing for an interruption of no more than 90 continuous minutes. Any nonzero value for beats per minute contributed to meeting the HR criterion. An interruption was permitted to allow for periods of wear, during which continuous heart rate measurement was not possible, such as when positioning of the arm caused the device to make poor contact with the skin, or at times of non-wear, such as during water activities. The HR criterion enables inclusion of participants whose recorded activity levels are low despite wearing the Fitbit for many hours, but excludes participants who wore the device only during exercise sessions. We defined a valid wear week as a period of seven consecutive days containing ≥4 days each meeting the HR criterion, since 3–5 valid wear days reliably predicts habitual MVPA in adults [47], though other studies have recommended as few as three of seven days [21].

Step count (SC) criterion. The second “step count” (SC) criterion defined a valid wear day as any day with ≥1000 recorded steps. We selected a 1000-step minimum based on an approximate mid-range of step count thresholds identified in several WAM-based intervention studies [24–28]. We also conducted preliminary analysis of daily step count and heart rate readings of 30 randomly selected FIReWoRk participants (15 with daily step counts of 500–1000 and 15 with 1000–2000). We found that participants with at least 1000 steps more consistently had continuous heart rate readings throughout the day, while participants with less than 1000 steps more often had heart rate data that were interrupted or incomplete. Opposite to the HR criterion, the SC criterion enabled inclusion of participants who wore the device only during exercise sessions, but excluded participants who, despite continuous wear, had recorded activity below the 1000-step threshold (such as those who were highly sedentary or performed predominately non-ambulatory activity). We also defined a valid wear week for the SC criterion as a period of seven consecutive days containing ≥4 days each meeting the 1000-step threshold. Some studies have suggested that a ≥3-day wear week is also an appropriate threshold for weekly step outcomes [48].

### 2.5. Definitions of Physical Activity Variables

To be included in PA variable creation, participants could not have zero data for day-level steps or minute-level heart rate data (thus, activity intensity data) during their first 100 full days of the intervention. We chose this minimum for inclusion because, based on known reasons for zero data after baseline, we could not assume data were missing at random. We focused on participants’ first 100 days in the intervention because we were interested in piloting pre–post assessments of PA outcomes that reflected change in PA at 3 months/13 weeks. We also wanted to capture change prior to increasing participants’ PA goal from 75 to 150 min per week. One hundred days encompassed their 3-month visit date as well as the 10-day buffer participants were given to complete their visit. We focused on PA outcome variables commonly reported in PA intervention studies. For the following variables, we derived two versions of each by applying the HR or SC criterion. We performed initial processing of Fitabase data in R open-source software version 3.6.2 (RStudio, Boston, MA, USA).

Valid days of wear in first 100 days. We defined the time period for valid wear days as starting on the first full day after the participant’s baseline visit date and proceeding until 100 consecutive days were reached. The number of valid wear days was the total of all days the participant met the respective wear criterion in the first 100 days. We also calculated valid wear as a percentage of valid days in 100.

Mean daily steps and active minutes in first 100 days. Mean steps per day and mean active minutes per day were the total number of steps or minutes for all valid days, divided by the number of valid days in the first 100 days. All participants who had at least four valid wear days in the first 100 days were included in the mean daily steps and mean daily active minutes calculations to limit the potential influence of days of activity that did not reflect habitual levels.

Mean daily steps and active minutes in Week 1 and Week 13. We defined Week 1 as the first seven consecutive days immediately following but not including the participant’s unique baseline visit date. A non-intervention run-in period to assess participants’ baseline PA was not conducted and participants did not receive their Fitbit until the first day of the intervention; therefore, assessment of pre-intervention PA levels was not possible. We defined Week 13 as the seven consecutive days leading up to, but not including, the participant’s 3-month visit date. Among the 22.5% of participants who did not complete their 3-month visit, we projected a 3-month visit date from their baseline date in order to approximate Week 13. We created indicators of whether a participant’s Week 1 and/or Week 13 were valid (i.e., ≥4 days meeting HR and/or SC criteria for a valid wear day). For participants with valid wear weeks in Week 1 and Week 13, we defined mean daily steps and mean daily active minutes as the total number of steps or minutes on all valid days, divided by the number of valid days per week. We also calculated total steps per week and total active minutes per week to approximate whether someone may be meeting PA guidelines in Week 1 and Week 13. For participants who had seven valid wear days, we summed daily steps and active minutes. For participants who had four, five or six valid wear days, we summed mean daily steps and active minutes across seven days (e.g., a mean of 20 active minutes per day across five valid days would be summed across seven days to approximate 140 weekly active minutes, e.g., [22]).

### 2.6. Analytic Sample

Of 668 total participants enrolled in the FIReWoRk Study, we included 616 participants who completed the first 100 days of the intervention prior to the COVID-19 stay-at-home orders implemented in March 2020, so that their data were not likely to be impacted by the COVID-19 pandemic. We then excluded participants with no Fitbit data recorded (i.e., either missing or zero step values) during their first 100 days and report known reasons for absent data. Among participants with at least some activity recorded during their first 100 days, we defined three analytic subsamples: those who had a valid Week 1 and Week 13 based on the (1) HR criterion only, (2) SC criterion only, and (3) both HR and SC criteria. We used participant ID to merge demographic and health-related information collected at a baseline survey interview with participants’ first 100 days of activity data from Fitabase. For baseline survey items, we replaced missing values (≤10 of 616 observations missing for any given variable) with the mode for categorical variables and the mean for continuous variables. We consulted staff notes from study visits to inform reasons for absent Fitabase accounts and/or Fitbit data.

### 2.7. Quantitative Analyses

**Figure 1.**
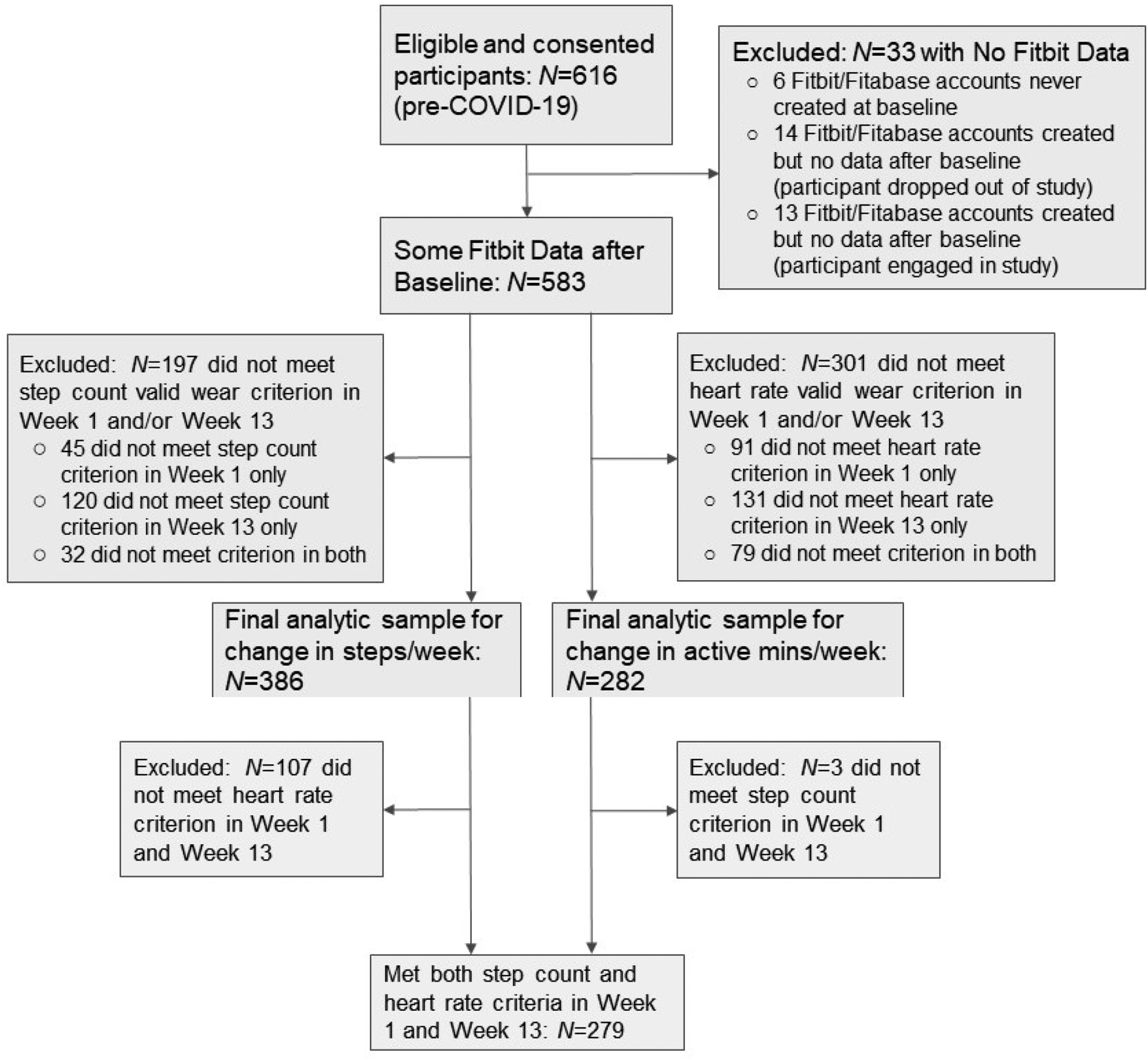
Flow diagram for analytic samples.

To describe the flow of participants into the three analytic subsamples, we calculated the number of participants that met HR and/or SC criteria for valid wear weeks and the frequencies of their known reasons for exclusion (see Figure 1). To explore differences in baseline demographic and health-related characteristics between groups meeting vs. not meeting SC and/or HR criteria for valid wear weeks, we conducted tests of significance. For categorical variables, we reported counts and frequencies and conducted nonparametric two-sample z-tests of proportions using the prtest function in Stata (StataCorp LLC, College Station, TX, USA). For normally distributed continuous variables, we reported means, standard deviations, and ranges and conducted unpaired two-sample t-tests assuming equal variances. For skewed distributions, we reported the median and interquartile range and employed the Mann–Whitney U test for comparison. We conducted all tests with a significance level of *α* = 0.05. Based on our experiences with FIReWoRk participants, we expected that more participants would meet the SC criterion than would meet the HR criterion for valid wear days and weeks. As such, we expected that more differences in demographic and health-related characteristics would emerge between groups with valid vs. invalid activity monitor wear based on the HR criterion than based on the SC criterion (see Table 1). Given the exploratory nature of our study, we did not hypothesize a priori which characteristics would significantly differ between groups with valid vs. invalid wear. We also calculated means, standard deviations, and ranges for PA outcomes commonly reported in behavioral intervention studies. We then compared and contrasted differences in outcomes between groups with varying availability of valid data for days (first 100 days) and weeks (Week 1 and 13) based on the HR and SC criteria (see Table 2).

We hypothesized that comparing SC-derived mean daily steps and active minutes to HR-derived mean daily steps and active minutes among participants meeting both criteria would yield adequate concordance between measures. To examine concordance, we calculated mean daily steps and active minutes for each Week 1 and Week 13 among participants with valid wear weeks for both criteria. We then employed concordance correlation coefficients (CCC) for repeated measures to take into account the repeated week that steps and active minutes were recorded per participant (see Table 3) [49]. We interpreted CCC values >0.80 as indicative of good concordance [50]. We analyzed concordance using the rm_ccc macro in SAS version 9.4 (SAS Institute Inc., Cary, NC, USA) [49].

### 2.8. Content Analysis

To explore issues experienced by participants that likely accounted for them not meeting one and/or two definitions of wear criteria, we conducted a content analysis of Fitbit issues documented by research staff at baseline, 1-, 2-, and 3-month study visits, as well as participant responses to 6-month exit interview questions assessing the acceptability of the Fitbit as a component of the weight-loss intervention. Approximately half of all FIReWoRk participants completed an exit interview on Fitbit acceptability. Participants who did not complete the interview either declined due to lack of time, had incomplete follow-up study visits, or reported not using the Fitbit during the study. One author identified initial categories of prevalent issues from research staff notes (MR) and openended exit interview responses (LG) and applied a code defined for each category to all similar responses until all responses were coded. A research assistant served as an independent second reviewer, applying the defined categories to uncoded responses, and identified any additional codes. A third reviewer resolved discrepancies in the coded data (SO). We then calculated frequencies of responses in each category to characterize issues regarding Fitbit use commonly reported by participants.

### 2.9. Qualitative Interview Methods

Participant recruitment and purposive sampling. To understand low-income, racially/ethnically diverse FIReWoRk participants’ experiences using the Fitbit for weight loss, we conducted in-depth, semi-structured interviews in June and July 2020. A pragmatic qualitative design structured the qualitative approach [51]. Pragmatic designs seek to generate practical, actionable qualitative findings. Consistent with this aim, we sought to elicit participants’ experiences with the Fitbit that we could apply in future research or clinical practice [51]. We purposively sampled a subset of FIReWoRk participants who completed the 12-month randomized controlled trial. To be eligible for an interview, participants (1) had been enrolled in the FIReWoRk study at one of the NYC intervention sites, (2) had completed the entire 12-month behavioral intervention, and (3) had consented to be contacted for future studies. FIReWoRk interventionists recommended participants who had recently completed the trial, and we contacted former participants to describe the research question and invite them to participate. We prioritized outreach to potential interviewees based on several pre-specified strata: gender (male or female), preferred language (English or Spanish), level of engagement with the Fitbit device during the trial (high or low), and intervention arm (goal-directed incentives, outcome-based incentives, or resources only). Interview participants tended to be among the more engaged in the FIReWoRk cohort and did not receive compensation for completing the interview.

Data collection. We developed a semi-structured interview guide to elicit participant perspectives on the Fitbit tool as a component of the behavioral intervention while still enabling flexibility in participant responses. FIReWoRk participants’ preliminary PA self-monitoring adherence data and exit interview feedback about the Fitbit, along with review of the human–computer interaction literature, informed the development of the interview guide by one author (LG), with input from two authors (SO and MJ). Three authors (LG, MR, and CH) conducted all interviews after completing qualitative interviewing training from an experienced qualitative researcher and conducting practice interviews with members of the research team. All interviewers had some prior experience conducting qualitative research, and one interviewer (CH) had existing relationships with participants from her role as a FIReWoRk interventionist.

Interviews lasted between 20 and 85 min and we conducted them using a secure video-conferencing software. However, not all participants utilized video. The team observed that audio-only interviews did not differ substantially from video interviews in content or duration. Audio recordings of the interviews were professionally transcribed verbatim. Spanish language interviews were simultaneously transcribed and translated into English transcripts. One bilingual interviewer (LG) listened to audio of Spanish interviews and checked translations for accuracy, finding no apparent issues with translation quality. All interviewers took notes following their interviews regarding the participant’s body language, tone, or any other details not able to be captured in the text transcript.

Data analysis. The three interviewers utilized team-based axial coding [52]. All three coders began with line-by-line, open coding of the same transcript, and met to compare open code labels and definitions, and to discuss areas of disagreement. Coding of a single transcript by all three coders was repeated three times, with all subsequent transcripts coded by at least two coders. With the first three transcripts, the coders discussed potential grouping or naming of codes based on open coding and the quantitative findings. Through consensus, the team constructed a preliminary codebook, which consisted of a code name and level, a definition, an exemplifying quote, and a link to corresponding quantitative variables when appropriate. After coding the initial three transcripts, we used the codebook to code subsequent transcripts. We treated the codebook as a living document, updating, expanding, or combining codes and definitions during regular team meetings as we analyzed more transcripts. We recoded transcripts coded earlier as we added new codes to the codebook.

We conducted open coding through Microsoft Word by combining documents to compare and contrast multiple investigators’ codes. Once the team reached consensus on a transcript, the transcript and codes were imported for analysis in ATLAS.ti version 8.4 (Scientific Software Development GmbH, Berlin, Germany). Analysis occurred simultaneously with interviewing, and the team regularly met to review codes, categories, and potential themes, and to determine coding saturation. We also utilized reflexive memoing [53] and team-based discussions to address potential biases in our approach to analysis. We attempted to achieve investigator triangulation [54] throughout the analysis by working with a team of individuals from different disciplines (nursing, medicine, and public health) and maintaining individual member’s independence in both data collection and analysis prior to team-based discussion.

## 3. Results

### 3.1. Prevalence of Participants with Valid Activity Monitor Data

As shown in Figure 1, 583 participants (94.6%) had some Fitbit data recorded in Fitabase during the first 100 full days following their unique baseline visit date. If a participant did not have data, either (1) the Fitabase/Fitbit account was never created at the baseline visit, (2) the participant’s account was created but they did not wear their device after baseline, or (3) the participant wore the Fitbit but device data were not synced/saved to the participant’s account before deletion. After exclusion of participants not meeting valid wear criteria in Week 1 and Week 13, 386 participants (62.7%) met the SC criterion for valid Week 1 and Week 13 data, and 282 (45.8%) met the HR criterion for valid Week 1 and Week 13 data. All except for three of the 282 participants who met the HR criterion also met the SC criterion. Thus, the SC criterion caught 98.9% of participants who met the HR criterion. Participants meeting the HR but not the SC criterion wore the monitor continuously, but did not accumulate ≥1000 steps on ≥4 days per week. In contrast, 107 more participants met the SC criterion than met the HR criterion. The HR criterion caught 72.3% of participants who met the SC criterion. The primary reason for this discrepancy was that the Fitbit device did not store minute-by-minute data for longer than one week; therefore, if the device was not synced with the Fitbit app, the data were not uploaded and the heart rate data were automatically deleted from the device.

### 3.2. Differences between Groups with and without Valid Wear Weeks

Table 1 describes differences between subsamples of participants with valid wear in Weeks 1 and 13 (≥4 valid days in both weeks) and invalid wear in Weeks 1 and 13 (<4 valid days in both weeks). Significantly more participants who reported that they did not use a smartphone had invalid wear weeks than those who reported smartphone use (*p* < 0.01). If they used a smartphone, significantly more participants who reported prior health app use had invalid wear weeks than those who reported no prior health app use (*p* < 0.05). Participants with invalid wear weeks had a slightly higher BMI on average than those with valid wear weeks (*p* = 0.02). These results were statistically significant when SC and HR criteria were applied to determine valid wear. When the HR criterion was applied, participants with invalid wear weeks were older on average than those with valid wear weeks (*p* = 0.04). Participants with invalid HR wear weeks more often identified as female, Hispanic, spoke Spanish at home, and preferred to speak Spanish during study visits, and less often reported working full-time or part-time, than those with valid HR wear weeks, though these differences were not significant (*p* = 0.07–0.10). When comparing the 107 participants with invalid HR wear weeks but valid SC wear weeks to the 282 participants with valid HR wear weeks, the HR criterion was significantly more inclusive of smartphone users (*p* < 0.001). The SC criterion seemed to be more inclusive of those who identified as female, spoke Spanish at home, were older in age, and lived in LA; however, these differences were not significant (*p* = 0.05–0.10).

### 3.3. Percentage Difference between PA Outcomes Using Step Count and Heart Rate Methods

As shown in Table 2, among the 583 participants with some Fitbit data recorded after baseline, there was a +18.5% difference in valid wear days in the first 100 days for the SC criterion compared to the HR criterion. In contrast to valid wear days, PA outcomes calculated for valid days (mean daily steps and mean daily active minutes) or valid weeks (total steps and total active minutes), on average, were higher when the HR criterion was applied compared to when the SC criterion was applied. For instance, among participants with at least 4 valid days recorded in the first 100 days, the percentage difference in HR-derived mean daily steps was +2.9% compared to the SC method. As a consequence, HR-derived absolute change in mean daily steps from Week 1 to Week 13 (*N* = 282 with valid wear weeks) was +42.3% different compared to SC-derived absolute change in mean daily steps (*N* = 386 with valid wear weeks).

### 3.4. Concordance between PA Outcomes among Participants Meeting Both Criteria

As shown in Table 3, among the 279 participants meeting both SC and HR valid wear week thresholds in Week 1 and Week 13, concordance between mean daily steps and active minute outcomes for weeks derived from SC vs. HR criteria was very high (CCC = 0.99, 95% CI = 0.99, 0.99). While we anticipated only a moderate correlation between number of valid days of data in the first 100 days when the SC vs. HR criteria were applied (CCC = 0.60 95% CI = 0.53, 0.66), Week 1 and Week 13 mean daily steps and active minute measures derived from each criterion were highly comparable.

### 3.5. Barriers to Wear from Research Staff Notes

Among 87 participants for whom the FIReWoRk research staff recorded notes describing barriers to Fitbit use that participants reported at baseline, 1, 2, and 3-month visits), we identified six issues repeatedly mentioned (in order from most to least frequent): (1) syncing and network issues (33.7%), (2) broken or misplaced device (18.9%), (3) lack of smartphone access (18.9%), (4) accuracy of recorded activity (13.7%), (5) trouble downloading Fitbit app (9.5%), and (6) barriers to keeping the device charged (5.3%). (See Supplemental Table S1a for category definitions and examples of issues from each category.)

### 3.6. Barriers to Wear from Intervention Exit Interviews

Among 364 FIReWoRk participants who completed an exit interview and reported using the device during the study, the majority (97.3%) said the Fitbit was ‘extremely’ (80.5%) or ‘somewhat’ (16.8%) helpful in successfully losing weight. Far fewer participants reported issues with using the device. Of the 364 respondents, 115 (31.6%) reported something they did not like about the device when asked to “Tell me about your experience with the Fitbit”. We identified 17 categories among 126 dislikes. The most frequently reported dislikes (11.1% of total) were that the band was uncomfortable to wear, the device was not user friendly/hard to understand, and it did not seem to track their perceived activity. The next most commonly reported dislike (8.7%) was preferring not to wear the device all the time/every day or only wearing it for the study. The third most common dislikes (7.9%) included issues with connecting or syncing the device to their smartphone, running out of battery or needing to charge it often, and a wrist band that broke (See Supplemental Table S1b for category definitions and examples of issues from each category).

### 3.7. Findings from Post-Study In-Depth Interviews

We interviewed 16 participants who completed the FIReWoRk Study, including 10 participants who identified as female, five who identified as male, and one who identified as non-binary. Five of the 11 participants were interviewed in Spanish per their preference. While the team had planned to recruit an equal number of Spanish-speaking participants, the no-show and refusal rates of Spanish speakers were higher than those of English speakers. We observed no apparent differences in themes between Spanish and English speakers. Four participants had reported lower vs. higher Fitbit engagement during the study. Finally, the three arms of the behavioral trial were nearly equally represented, with five participants from the resources only arm, five from the outcome-based incentives arm and six from the goal-directed incentives arm. Interview participants ranged in age from 24 to 70 years.

After 14 interviews, the team reached coding saturation, with subsequent interviews generating no new codes or categories. Three themes emerged across the interviews that were relevant to controllable and uncontrollable barriers to Fitbit wear: (1) technology issues, (2) physical device issues, and (3) negative emotional responses to self-monitoring.

#### Technology issues.

Participants across the interviews described issues with the Fitbit device or application (app) technology that inhibited their abilities to track and upload PA data for both self-monitoring and study data collection. These technology concerns included participants’ access to a compatible smartphone to enable syncing, failure of the device to upload data to the app, or the device simply failing to retain charge over time. Participants also expressed frustration in the Fitbit’s inability to accurately capture their perceived PA, including a participant who worked as a bus driver and found the Fitbit miscalculating his driving as steps. He shared, “because a bus doesn’t move very fast … it will think that I’m speed walking or lightly jogging. So, the first few months that I had it, it was driving me nuts ‘cause I kept getting notifications, congratulations. You’ve done 50,000 and I really hadn’t.”

Participants with limited or no access to the technology that was needed to utilize the Fitbit were unable to fully engage with the device and the app. For those without a smartphone or with an older phone incompatible with the Fitbit, self-monitoring was difficult or unachievable. One participant shared his frustrating experiences trying to self-monitor with an older phone, “When I joined the study, I had an Android and I didn’t have this one. I had a J3, which is a very old one. After a while, it wouldn’t sync with it and I had trouble syncing with it.”

Self-identified “older” participants expressed issues using Fitbit technology and reported technology literacy barriers that impacted their engagement with PA self-monitoring. Technology literacy issues were focused mainly on the Fitbit app, which participants described as “confusing.” One participant described her attempts to fully utilize the Fitbit and app as similar to her struggles with using a computer, “I can’t work so much stuff on my computer, I don’t understand and I only do a small bit of what I can grasp. So, it’s another piece of equipment of computer brain that I don’t understand.”

#### Physical device issues.

In addition to technology barriers, several interview participants shared issues they experienced while wearing the Fitbit that inhibited consistent wear. The Fitbit wristband, in particular, caused them problems. Participants shared frustration with the strap breaking or being uncomfortable, especially when sleeping. One participant described not using the Fitbit regularly because of how uncomfortable it was to wear while sleeping, and that she preferred “to sleep without anything.” Other participants described a need to replace a broken strap, which one participant described as “a bit vulnerable.” Participants described having to wait until their next study visit to replace the band or needing to pay for a replacement band after completing the study in order to continue to use the Fitbit for self-monitoring.

#### Negative emotional responses to self-monitoring.

Interview participants described a range of emotional responses to PA self-monitoring which either encouraged or discouraged wear. While some participants described enjoying the Fitbit’s alerts and reminders and were motivated by seeing progress in their PA achievements, others experienced alert fatigue or had a negative emotional response to wearing the Fitbit. Participants described feelings of self-doubt or disappointment with their daily activity data, or frustration with the frequency of Fitbit alerts and messages, which impacted their likelihood of wearing the Fitbit. One participant described her weight-loss journey as a “roller-coaster” and shared that tracking active minute data and working toward Fitbit goals became fatiguing over time. She described a shift in her behavior from exercising for wellness to focusing too heavily on achieving the “credit” monitored by the Fitbit. She eventually stopped wearing the Fitbit, finding the pressure to achieve the credit more stressful than trying to lose weight without the Fitbit.

Alerts were an additional stressor that led to participant frustration with regular Fitbit use. One participant shared how the messaging of the alerts was demotivating, “I don’t like the way it tells me I didn’t do as much as I did the week before. I wish I could just stop it from saying that. That has nothing to do with me.” Some participants described frequent alerts, even alerts celebrating meeting an activity goal, as “annoying” and preferred looking at their data unprompted. For some, changing alert settings solved this issue, but for others who had more difficulty with the technology or did not have access to the app to change their settings, alarm fatigue and frustration persisted.

## 4. Discussion

As expected, more participants had valid wear in Week 1 and Week 13 based on the SC criterion than the HR criterion. The 62.7% of valid wear days for the HR criterion was lower in our sample than in comparable interventions using ≥10 h/day of minute-level data to determine Fitbit wear (80.4–88.1%) [55,56]. Few differences in demographic and health-related characteristics emerged between participants with and without valid wear weeks, suggesting other explanatory factors may be relevant in predicting valid wearable activity monitor (WAM) data. Significant between-group differences emerged with regard to level of smartphone and health app use prior to the intervention, which could have implications for technology access and literacy as moderators of the effectiveness of weight-loss interventions with a WAM component. It is possible that some participants continued to engage with a familiar app rather than with the Fitbit app during the intervention, or were entering the study already disillusioned with the efficacy of similar apps for weight loss. Findings of significant differences in BMI between groups, combined with our understanding of participants’ experiences with the Fitbit from qualitative inquiry, may be attributed to greater band discomfort, and therefore, less continuous wear, among those with a higher BMI. We expected that a greater number of significant differences would emerge between groups with invalid vs. valid activity monitor wear determined using the HR criterion than using the SC criterion. However, only age was additionally significantly different between groups for the HR method. Older participants had fewer days of valid wear, a finding contrary to prior research in which older participants were more adherent to PA apps [40]. Age-related barriers to regularly syncing the device to a smartphone app via Bluetooth likely contributed to this finding, illustrated by older participants’ quotes that acknowledged low smartphone literacy or using outdated technology. Significant differences in valid wear by Hispanic ethnicity and Spanish language, as well as saturation of qualitative codes related to cultural or language barriers, were not confirmed in our study. Larger scale qualitative inquiry of Hispanic patients with obesity may identify unique barriers to PA data collection and self-monitoring using WAMs. Our study demonstrated higher frequencies of Spanish speakers in invalid wear groups, which is supported anecdotally by the various personal reasons for not wearing the Fitbit (e.g., taking it off during work) that participants shared with FIReWoRk interventionists.

PA goals encouraged in the FIReWoRk study reflected public health recommendations that adults accumulate ≥150 min of moderate-intensity PA, or ≥75 min of vigorous-intensity PA, per week to maximize health benefits [46]. However, after realizing the memory limits of the Fitbit device, the research team made adaptations early in the study to translate the weekly active minutes goal to a weekly step count goal for participants who were unable to sync their device regularly. Otherwise, these participants would not have had data available during study visits for the interventionist to use to provide feedback on PA goal progress during the previous month. The total daily volume of ambulatory physical activity associated with meeting MVPA recommendations is at least 7000–8000 steps per day [57]. Recent evidence suggests that less strenuous light-intensity PA (LPA), such as walking at a rate less than 3000 steps in 30 min, and generally reducing sedentary behaviors, confers health benefits [58,59]. In free-living conditions, consumer-grade WAMs may yield LPA and step outcomes more accurately than MVPA outcomes [12,60–62]. Thus, LPA and steps per day may be preferred in some behavioral interventions as a meaningful measure of PA change, particularly in populations likely to have high levels of invalid WAM data due to the absence of minute-by-minute-level data needed for calculating MVPA outcomes.

In this study, one in three participants still did not have valid data based on the SC criterion to determine total steps in Week 1 and Week 13. Therefore, applying a SC criterion to daily step-based PA outcomes in future studies likely is not sufficient to address the substantial uncontrollable technology issues identified in this sample of low-income, majority Latina adults with obesity. Best practices exist for increasing “wear adherence” [42,47,63]; however, the concept of participant wear compliance seems a bit of a misnomer when deploying consumer-grade WAMs. Even in cases of adherent Fitbit wear, uncontrollable barriers to data collection and storage also contributed to high levels of invalid data. One’s ability to sync device data to a database at regular intervals was crucial, because even though step data were saved for 30 days, if a participant had missed one monthly visit where the interventionist synced the device, it resulted in a loss of 30 days of step data. If a participant was not able to sync their own device, they also were not able to interact with their data on the Fitbit app, potentially diminishing its effectiveness as a behavior change tool. Additional barriers included but were not limited to lack of access to compatible and up-to-date smartphone technology with enough functionality and memory for app use, a Fitbit app interface that was not user friendly to some, and wristbands that caused skin irritation or discomfort. The FIReWoRk study team mitigated these barriers to the extent possible during the intervention. For instance, interventionists provided instruction at baseline on charging and syncing the device and interacting with the participant’s own Fitbit app, and worked to resolve any issues that arose during participation. Interventionists also offered a Fitbit to borrow when a device issue could not be resolved, as well as replacement bands of various sizes and of a different material when participants reported band irritation or discomfort. The FIReWoRk study did not exclude participants who did not own an intervention-compatible smartphone. Future interventions in low-income, racially/ethnically diverse adults with obesity may consider ensuring Wi-Fi and/or cellular data service is sufficient in study visit locations and lending smartphones to participants without regular access to one, or when service is interrupted.

When assessed at baseline, indicators of income such education level, employment status, health insurance, food security status, financial wellbeing, and neighborhood income were not significantly different between groups with and without valid wear weeks. Psychological indicators of behavioral engagement such as PA self-monitoring in the previous month, intrinsic motivation to self-monitor, intention to be physically active in the next month, and self-reported MVPA levels were not significantly different between groups with and without valid wear weeks. These findings suggest that such baseline characteristics likely do not predict whether someone ultimately accumulates valid wear data. Nonetheless, there may be a need to address the emotional toll of WAM-based PA self-monitoring over several months, which emerged as a theme in post-intervention interviews. Considering prevalent application of WAM technology in weight-loss interventions, it is important to understand how WAMs can best facilitate self-monitoring [55], an evidence-based weight management strategy [43,64,65]. Establishing protocols for research staff to deactivate default notifications and allowing the participant to ‘opt-in’ to such messages if preferred may prevent the alert fatigue that participants described. In addition, testing and standardizing messages that interventionists can relay when a participant expresses concerns that the device was not adequately capturing their perceived level of activity may benefit future PA interventions. Understandably, when participants feel they are making an effort, but not getting “credit” for their PA, it can be demotivating. Interventionists may be able to respond to such concerns by reframing goals in terms of a participant’s unique intrinsic motivators for changing PA behavior.

### 4.1. Strengths and Limitations

Fitbit technology is a popular behavior change modality that allows interventionists to verify participants’ PA goal attainment and provide timely feedback. Several methodological limitations exist to PA assessment with Fitbit data. Existing accelerometer data collection and processing protocols for PA research originate from surveillance studies. Some approaches may not be advisable for use in intervention research. For example, measurement protocols often assume participants are not reactive to wearing the monitor, whereas most WAMs provide feedback intended to alter behavior. Interpolating missing data with mean daily steps or minutes from valid wear days may overestimate weekly PA outcomes if the participant only wears the device during activity. Equating four and seven valid wear days may misrepresent the PA levels of groups if individuals with more valid wear days are more active due to wearing the device more often. Using 3-day instead of 4-day wear thresholds in future studies may improve the inclusivity of analytic samples. This study provides unique guidance to PA researchers interested in using Fitbit technology on how to potentially decrease missing data and increase inclusivity in their analytic samples. Some of these recommendations may lose relevance as WAM devices and algorithms improve.

Integration of qualitative data into this study provided in-depth insight into the quantitative prevalence of valid wear, and potential barriers to long-term Fitbit engagement. We incorporated several data mixing elements, including (1) using quantitative data to inform the post-study qualitative interview schedule and selection of interview participants, (2) quantifying the qualitative data from research staff notes and participant exit interviews, and (3) integrating quantitative and qualitative findings in the discussion. Qualitative inquiry also allowed us to explore unique barriers to WAM use in an understudied patient population. Research staff notes and exit interview responses were not available for all participants; therefore, the barriers identified may not represent all barriers experienced by participants. While the team achieved coding saturation in data collected from in-depth interviews, this study did not address thematic or meaning saturation [66], since coding saturation was sufficient to generate practical findings that are applicable to future clinical research. Due to staff resource limitations, we conducted qualitative interviews with participants from the NYC and not the LA study sites, which may limit the generalizability of our qualitative findings; however, we prioritized an in-depth understanding of fewer participants over broad generalizability of these findings.

### 4.2. Future Research Directions

The field of wearable accelerometry is developing rapidly. Reviews and meta-analyses are needed every few years in order to identify and update best practices on data collection and processing criteria [67]. Researchers should continue to develop tailored strategies to improve device functionality, maximize participant engagement, and minimize missing data [42]. Since data completeness inevitably permeates continuous measurement of PA over time, future studies are needed that evaluate various approaches to correct for missing data in analysis [23]. Finally, few WAM interventions incorporate thorough qualitative analysis of the WAM features intervention participants prioritize [68]; thus, qualitative and mixed methods research is necessary to address the specific needs of low-income, racially/ethnically diverse populations.

## 5. Conclusions

There are a lack of consistent descriptions of wear and data processing decisions to guide researchers employing consumer-grade WAMs to measure PA outcomes in behavioral interventions. Through quantitative and qualitative inquiry into weight-loss intervention participants’ first 100 days of Fitbit activity, we learned that applying standard definitions of activity monitor wear in the interest of valid MVPA outcomes would diminish the inclusivity of our analytic sample, and thus, the generalizability of our findings. The Fitbit device was not particularly sensitive in measuring daily MVPA over time, and applying activity monitor wear standards used in MVPA surveillance studies was inadvisable, in part due to insufficient capacity of the device to store data when the device could not be synced regularly. Such technological barriers may disproportionately affect intervention participants who are older in age, have a higher BMI, and have lower smartphone technology access and literacy. Applying a step count criterion (≥1000 steps/day) to mean daily steps rather than a minute-by-minute heart rate criterion (≥10 h/day) to mean daily MVPA minutes required less data processing and excluded fewer participants, particularly older Latinas, from outcomes analysis while yielding highly concordant PA outcomes. Future research should standardize robust definitions of valid activity monitor data that are also inclusive of various user groups, so that researchers can apply them consistently in intervention studies.

## Supplementary Material

Supplemental Figure 1 and Table 1

## Figures and Tables

**Table 1.** Differences between groups with and without valid activity monitor wear in Weeks 1 and 13.

	All Participants *N* = 616	Step Count (SC) Wear Criterion			Heart Rate (HR) Wear Criterion			Invalid HRbut Valid SC Wear Weeks *N* = 107	*p*-Value^a^
		Participants with Invalid SC Wear Weeks *N* = 230	Participants with Valid SC Wear Weeks *N* = 386	*p*-Value	Participants with Invalid HR Wear Weeks *N* = 334	Participants with Valid HR Wear Weeks *N* = 282	*p*-Value		
*Categorical Variables*	*N* (%)	*N* (%)	*N* (%)		*N* (%)	*N* (%)		*N* (%)	
**Gender**									
Female	499 (81.0)	190 (82.6)	309 (80.1)	0.49	279 (83.5)	220 (78.0)	0.10	92 (86.0)	0.10
Male	117 (19.0)	40 (17.4)	77 (20.0)	0.73	55 (16.5)	62 (22.0)	0.45	15 (14.0)	0.49
**Race/Ethnicity**									
Hispanic	451 (73.2)	175 (76.1)	276 (71.5)	0.28	256 (76.7)	195 (69.2)	0.07	84 (78.5)	0.11
Non-Hispanic Black	88 (14.3)	30 (13.0)	58 (15.0)	0.80	41 (12.3)	47 (16.7)	0.56	11 (10.3)	0.60
Non-Hispanic White	38 (6.2)	11 (4.8)	27 (7.0)	0.80	16 (4.8)	22 (7.8)	0.71	5 (4.7)	0.81
Other	39 (6.3)	14 (6.1)	25 (6.5)	0.96	21 (6.3)	18 (6.4)	0.99	7 (6.5)	0.99
**Immigrant to United States**									
Yes	394 (64.0)	148 (64.4)	246 (63.7)	0.89	217 (65.0)	177 (62.8)	0.65	71 (66.4)	0.59
**Spanish Spoken at Home**									
Yes	427 (69.3)	164 (71.3)	263 (68.1)	0.49	244 (73.1)	183 (64.9)	0.07	81 (75.7)	0.08
**Spanish Spoken at Study Visit**									
Yes	289 (46.9)	118 (51.3)	171 (44.3)	0.24	173 (51.8)	116 (41.1)	0.07	56 (52.3)	0.16
**Employment Status**									
Working full- or part-time	289 (46.9)	103 (44.8)	186 (48.2)	0.58	141 (42.2)	148 (52.5)	0.09	38 (35.5)	0.06
Unemployed or looking for work	133 (21.6)	46 (20.0)	87 (22.5)	0.74	69 (20.7)	64 (22.7)	0.78	25 (23.4)	0.94
Keeping house or raising children	134 (21.8)	51 (22.2)	83 (21.5)	0.92	83 (24.9)	51 (18.1)	0.36	33 (30.8)	0.17
Retired	60 (9.7)	30 (13.0)	30 (7.8)	0.51	41 (12.3)	19 (6.7)	0.51	11 (10.3)	0.73
**Education**									
8th grade or less	129 (20.9)	63 (27.4)	66 (17.1)	0.16	84 (25.2)	45 (16.0)	0.23	22 (20.6)	0.64
Some high school	83 (13.5)	25 (10.9)	58 (15.0)	0.62	46 (13.8)	37 (13.1)	0.93	22 (20.6)	0.45
High school grad or equivalent	148 (24.0)	61 (26.5)	87 (22.5)	0.58	83 (24.9)	65 (23.1)	0.80	22 (20.6)	0.81
Some college	160 (26.0)	51 (22.2)	109 (28.2)	0.42	76 (22.8)	84 (29.8)	0.32	26 (24.3)	0.24
4-year college grad or higher	96 (15.6)	30 (13.0)	66 (17.1)	0.61	45 (13.5)	51 (18.1)	0.54	15 (14.0)	0.71
**Marital Status**									
Married	234 (38.0)	85 (37.0)	149 (38.6)	0.81	127 (38.0)	107 (37.9)	0.99	42 (39.3)	0.87
Separated or divorced	121 (19.6)	46 (20.0)	75 (19.4)	0.94	69 (20.7)	52 (18.4)	0.75	24 (22.4)	0.68
Widowed	25 (4.1)	12 (5.2)	13 (3.4)	0.82	16 (4.8)	9 (3.2)	0.85	4 (3.7)	0.96
Never married	236 (38.3)	87 (37.8)	149 (38.6)	0.90	122 (36.5)	114 (40.4)	0.54	37 (34.6)	0.53
**Health Insurance**									
Public insurance	526 (85.4)	192 (83.5)	334 (86.5)	0.35	283 (84.7)	243 (86.2)	0.63	94 (87.9)	0.67
No insurance	87 (14.1)	36 (15.7)	51 (13.2)	0.74	48 (14.4)	39 (13.8)	0.95	12 (11.2)	0.50
Unknown insurance	3 (0.5)	2 (0.9)	1 (0.3)		3 (0.9)	0 (0.0)		1 (0.93)	
**Smartphone Use**									
Yes	539 (87.5)	189 (82.2)	350 (90.7)	0.004**	273 (81.7)	266 (94.3)	<0.001***	86 (80.4)	<0.001***
**Health App Use**									
Yes	109 (17.7)	48 (20.9)	61 (15.8)	0.49	58 (17.4)	51 (18.1)	0.92	11 (10.3)	0.53
No	443 (71.9)	147 (63.9)	296 (76.7)	0.005 **	225 (67.4)	218 (77.3)	0.02 **	79 (73.8)	0.53
Not applicable	64 (10.4)	35 (15.2)	29 (7.5)	0.34	51 (15.3)	13 (4.6)	0.31	17 (15.9)	0.33
**Activity Monitor Wear**									
Yes	35 (5.7)	15 (6.5)	20 (5.2)	0.87	17 (5.1)	18 (6.4)	0.87	2 (1.9)	0.80
No	84 (13.6)	33 (14.4)	51 (13.2)	0.88	50 (15.0)	34 (12.1)	0.71	19 (17.8)	0.57
Not applicable	497 (80.7)	182 (79.1)	315 (81.6)	0.50	267 (80.0)	230 (81.6)	0.65	86 (80.4)	0.81
**Cigarette Smoking (past 30 days)**									
Yes	58 (9.4)	23 (10.0)	35 (9.1)	0.91	35 (10.5)	23 (8.2)	0.77	12 (11.2)	0.77
**History of Heart Problems**									
Yes	55 (8.9)	26 (11.3)	29 (7.5)	0.63	37 (11.1)	18 (6.4)	0.58	12 (11.2)	0.64
**History of Lung Problems**									
Yes	88 (14.3)	37 (16.1)	51 (13.2)	0.70	49 (14.7)	39 (13.8)	0.90	13 (12.2)	0.88
**History of Arthritis**									
Yes	189 (30.7)	78 (33.9)	111 (28.8)	0.46	106 (31.7)	83 (29.4)	0.73	30 (28.0)	0.88
**History of Bariatric Surgery**									
Yes	29 (4.7)	13 (5.7)	16 (4.2)	0.85	14 (4.2)	15 (5.3)	0.89	3 (2.8)	0.85
**Food Security Status**									
High or marginal	365 (59.3)	123 (53.5)	242 (62.7)	0.10	190 (56.9)	175 (62.1)	0.31	68 (63.6)	0.83
Low	182 (29.6)	80 (34.8)	102 (26.4)	0.22	104 (31.1)	78 (27.7)	0.62	26 (24.3)	0.73
Very low	69 (11.2)	27 (11.7)	42 (10.9)	0.92	40 (12.0)	29 (10.3)	0.83	13 (12.2)	0.86
**Clinic Site**									
Brooklyn, New York	194 (31.5)	86 (37.4)	108 (28.0)	0.16	111 (33.2)	83 (29.4)	0.57	26 (24.3)	0.61
BManhattan, New York	170 (27.6)	65 (28.3)	105 (27.2)	0.88	85 (25.5)	85 (30.1)	0.50	21 (19.6)	0.34
Los Angeles, California	252 (40.9)	79 (34.4)	173 (44.8)	0.12	138 (41.3)	114 (40.4)	0.89	60 (56.1)	0.05
Age (years)	48.7 (12.4)	48.4 (12.6)	47.3 (12.3)	0.29	48.7 (12.8)	46.6 (11.8)	0.04*	49.2 (13.1)	0.06
Body mass index (BMI)	38.0 (6.6)	38.8 (7.1)	37.5 (6.2)	0.02*	38.5 (6.8)	37.3 (6.3)	0.02*	38.4 (6.9)	0.14
Total physical activity MET-minutes per week^b^	922.0 (2504.0)	1055.0 (2689.0)	866.3 (2313.0)	0.63	792.0 (2574.0)	960.0 (2337.0)	0.39	693.0 (2330.0)	0.09
Physical activity behavioral intention	6.2 (1.2)	6.1 (1.2)	6.2 (1.2)	0.32	6.1 (1.2)	6.2 (1.2)	0.30	6.1 (1.3)	0.47
Intrinsic motivation for self-monitoring	1.4 (1.5)	1.5 (1.5)	1.4 (1.5)	0.42	1.4 (1.5)	1.5 (1.5)	0.41	1.3 (1.5)	0.24
Financial well-being score	55.8 (12.8)	55.3 (12.3)	56.0 (13.1)	0.51	55.5 (12.5)	56.0 (13.2)	0.63	55.6 (13.0)	0.79
Tract median household income	$34,626.1 (10,254.4)	$34,445.4 (8641.40)	$33,660.7 (8258.10)	0.26	$34,100.1 (8477.00)	$33,780.3 (8330.20)	0.64	$34,206.4 (8120.80)	0.65

a Compared to 282 participants with valid heart rate (HR) wear;

b Total physical activity MET-minutes/week assessed using the International Physical Activity Questionnaire short form, reported as median (interquartile range), and compared using Mann-Whitney U test;

* *p* < 0.05,

** *p* < 0.01,

*** *p* < 0.001.

**Table 2.** Physical activity outcomes among participants with valid wear based on Step Count or Heart Rate criterion.

Physical Activity Variables	Step Count Wear Criterion			Heart Rate Wear Criterion		
	*N*	M (SD)	Range	*N*	M (SD)	Range
Valid Days of Data in First 100 Days	583	75.7 (28.0)	0 to 100	583	62.7 (31.3)	0 to 100
**Steps**						
Mean Daily Steps in First 100 Days	571	8743.4 (3869.3)	1671.5 to 27,905.2	563	9000.3 (4006.7)	670.1 to 23,284.9
Mean Daily Steps in Week 1	506	9057.6 (4163.8)	1721.4 to 29,103.0	413	9525.3 (4378.2)	683.5 to 29,103.0
Total Steps in Week 1	506	63,403.5 (29,146.8)	12,049.8 to 20,3721.0	413	66,677.4 (30,647.5)	4784.5 to 20,3721.0
Mean Daily Steps in Week 13	431	9040.4 (4271.7)	1677.0 to 24,030.9	373	9448.1 (4456.8)	705.7 to 24,030.9
Total Steps in Week 13	431	63,283.1 (29,901.7)	11,739.0 to 16,8216.0	373	66,136.9 (31,197.4)	4940.0 to 168,216.0
Change in Mean Daily Steps from Week 1 to 13	386	−162.4 (3026.6)	−11961.7 to 12856.1	282	−249.4 (2868.7)	−11,961.7 to 12,856.1
**Active Minutes**						
Mean Daily Active Minutes in First 100 Days	NA	NA	NA	563	43.6 (40.9)	0 to 299.3
Mean Daily Active Minutes in Week 1	NA	NA	NA	413	47.0 (43.8)	0 to 299.7
Total Active Minutes in Week 1	NA	NA	NA	413	328.9 (306.9)	0 to 2098.0
Mean Daily Active Minutes in Week 13	NA	NA	NA	373	54.8 (55.5)	0 to 396.0
Total Active Minutes in Week 13	NA	NA	NA	373	383.4 (388.2)	0 to 2772.0
Change in Mean Daily Active Minutes from Week 1 to 13	NA	NA	NA	282	6.4 (44.4)	−160.1 to 271.4

Note: NA = Not Available due to heart rate data requirement for deriving active minutes.

**Table 3.** Concordance between Step Count- and Heart Rate-derived physical activity outcomes among participants with valid data.

Physical Activity Variables	Concordance Coefficient			
	*N*	CCC	SE CCC	95% CI
Valid Days of Data in First 100 Days	279	0.597	0.034	0.527–0.659
Mean Daily Steps in Week 1 and Week 13	279	0.988	0.001	0.986–0.990
Mean Daily Active Minutes in Week 1 and Week 13	279	0.990	0.001	0.988–0.992

## Abbreviations

WAM: wearable activity monitor
PA: physical activity
LPA: light-intensity physical activity
MVPA: moderate to vigorous-intensity physical activity
SC: step count
HR: heart rate
BMI: body mass index
