# Supplementary material for: Defining Valid Activity Monitor Data: A Multimethod Analysis of Weight-Loss Intervention Participants’ Barriers to Wear and First 100 Days of Physical Activity
Source: Informatics (MDPI). Author manuscript; Available in PMC 2022 Dec 15. (PMC9754231; doi:10.3390/informatics8020039)
Supplement: Supplemental Figure 1 and Table 1 [file NIHMS1715136-supplement-Supplemental_Figure_1_and_Table_1.pdf]

Supplemental Figure S1. Examples of Valid and Invalid Wear Days for Heart Rate Criterion.

Figure S1a. Example of a participant's ≥10 hours of heart rate data over 24 hours (valid wear day based on the heart rate criterion)

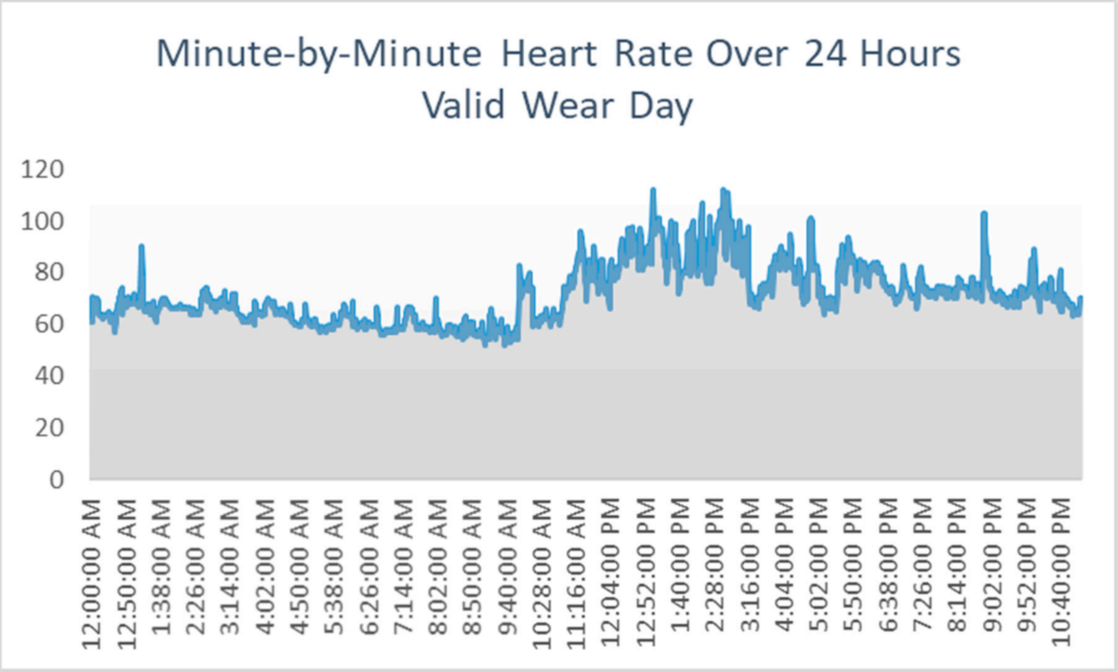

Figure S1b. Example of a participant's <10 hours of heart rate data over 24 hours (invalid wear day based on the heart rate criterion)

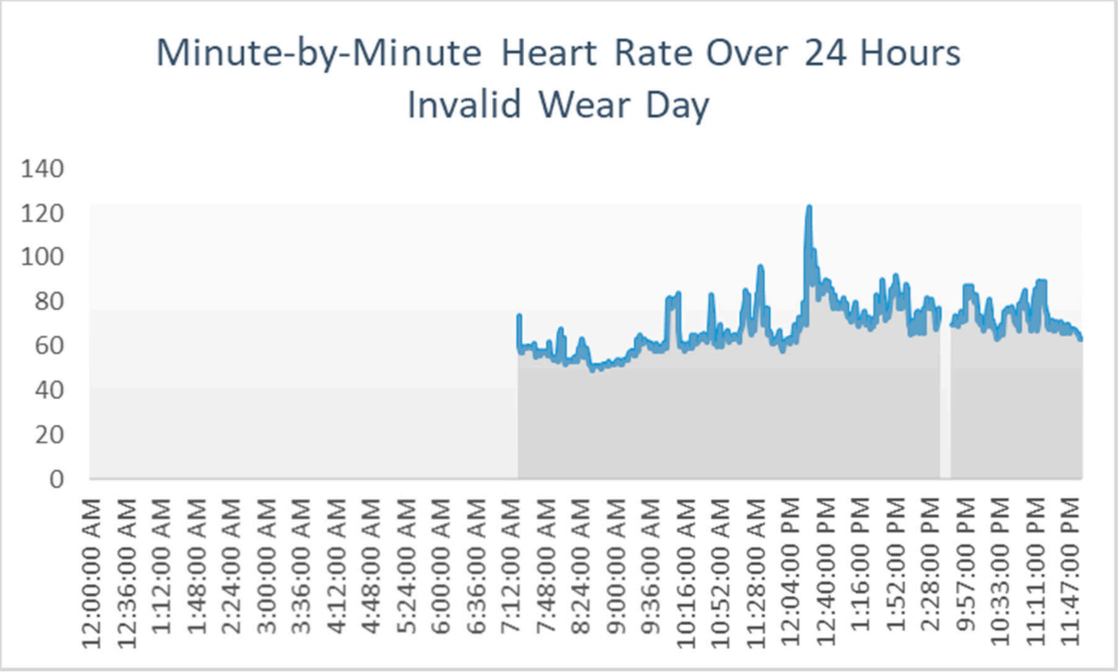

Orstad, S.L.; Gerchow, L.; Patel, N.R.; Reddy, M.; Hernandez, C.; Wilson, D.K.; Jay, M. Defining Valid Activity Monitor Data: A Multimethod Analysis of Weight-Loss Intervention Participants' Barriers to Wear and First 100 Days of Physical Activity. *Informatics* **2021**, *8*, 39. https://doi.org/10.3390/informatics8020039

Supplemental Table S1. Frequency of Barriers to Fitbit Wear Identified in Qualitative Data

**Table S1a. Barriers to Fitbit Wear Identified in Research Staff Notes* (87 participants)**			
**Issue Category**	**Definition**	**Examples**	**N (%)**
1. Syncing and network issues	Participants reported barriers to syncing the data stored on the device to their respective Fitbit account, leading to problems with consolidating the data even if physical activity was captured by the device.	"Has had a hard time getting Fitbit to sync for the last week. Only synced in room on different phone" and "Patient was given new Fitbit because her old one stopped syncing completely even after disconnecting it from her account. It did not want to re-attach to her account."	32 (33.7%)
2. Broken or misplaced device	Participants experienced physical malfunctions or misplacement of their device during at least some parts of the study.	"Patient can't find her Fitbit. I was unable to record any data" and "Pt was not wearing her Fitbit all month, it was dead and she thought it was not working."	18 (18.9%)
3. Lack of smartphone access	Participants reported not owning a smartphone. (While it is not necessary for a Fitbit user to have a smartphone, it facilitates downloading the Fitbit app, accessing additional features, and syncing the data.)	"Patient does not have smartphone." and "Fitbit wasn't syncing the first few weeks due to lack of smartphone. Now using her daughter's phone to sync."	18 (18.9%)
4. Perceived accuracy of recorded activity	Participants perceived device dysfunction with recording physical activity and steps, including dysfunction with their Bluetooth connection in recording their activity.	"The Fitbit had some malfunctions every once in a while, where it does not record her active min…" and "Old Fitbit was over marking active minutes and not marking some days…".	13 (13.7%)
5. Trouble downloading Fitbit application	Participants reported problems downloading the Fitbit application onto their smartphone.	"Did not have the Fitbit with her and could not download the application on her phone. Writes down her numbers in her journal but forgot it this visit." and "…Patient cannot download the Fitbit app onto the phone".	9 (9.5%)
6. Barriers to keeping the device charged	Participants experienced barriers to adequately charging the battery of their device	"Fitbit battery died two weeks after baseline and patient couldn't find the charger" and "Patient was out of the country due to family emergency for all of January. Had left Fitbit charger at home so no activity was recorded."	5 (5.3%)
**Total Count**			95

*Research staff notes examined from baseline, 1-, 2-, and 3-month study visits.

Orstad, S.L.; Gerchow, L.; Patel, N.R.; Reddy, M.; Hernandez, C.; Wilson, D.K.; Jay, M. Defining Valid Activity Monitor Data: A Multimethod Analysis of Weight-Loss Intervention Participants' Barriers to Wear and First 100 Days of Physical Activity. *Informatics* **2021**, *8*, 39. https://doi.org/10.3390/informatics8020039

Table S1b. Barriers to Fitbit Wear Identified in 6-month Exit Interviews (115 participants)			
**Issue Category**	**Definition**	**Examples**	**N (%)**
1. Interface difficulty	Participants reported difficulty with understanding how to use the device interface in an effective manner.	"I never figured out how to actually work it..." and "I didn't understand the stats or what the labels meant"	14 (11.1%)
2. Perceived accuracy of recorded activity	Participants perceived device dysfunction with recording physical activity and steps.	"It would sometimes stop counting my active minutes or refuse to work, so it was occasionally frustrating" and "I didn't like that it often times didn't track my active minutes even when I walked."	14 (11.1%)
3. Physically uncomfortable to wear	Participants reported feeling physically uncomfortable due to wearing the device.	"I felt it irritated the bones in my fingers" and "…the band made my skin break out."	14 (11.1%)
4. Preference for not wearing continuously	Participants reported not wearing the device continuously throughout the study or wearing just for the purposes of the study.	"I didn't like wearing it" and "I was wearing it for the study" and "I liked that it showed me how much I was doing but I didn't like wearing it all the time."	11 (8.7%)
5. Syncing and network issues	Participants reported barriers to syncing the data stored on the device to their respective Fitbit account, leading to problems with consolidating the data even if physical activity was captured by the device.	"…sometimes it would disconnect from my account and it was hard to reconnect" and "I had a little trouble staying connected with the Fitbit app."	10 (7.9%)
6. Barriers to keeping device charged	Participants experienced barriers to adequately charging the battery of their device or reported a shorter-than-expected battery life.	"The only thing I didn't like is that when the battery died it would die for real" and "I didn't like that my battery seemed to die a little too quick."	10 (7.9%)
7. Broken wrist band	Participants reported the device band broke.	"The band broke and fell off and lost the first Fitbit" and "I didn't like that the band broke off early."	10 (7.9%)
8. Device not waterproof	Participants reported not liking that the device was not waterproof.	"I hate that its not water proof" and "…I wish it was waterproof"	8 (6.3%)
9. Technology errors	Participants reported technical errors with their device, including that it stopped working.	"I didn't like that it froze and stopped working for me" and "Fitbit was a little glitchy."	6 (4.8%)

Orstad, S.L.; Gerchow, L.; Patel, N.R.; Reddy, M.; Hernandez, C.; Wilson, D.K.; Jay, M. Defining Valid Activity Monitor Data: A Multimethod Analysis of Weight-Loss Intervention Participants' Barriers to Wear and First 100 Days of Physical Activity. *Informatics* **2021**, *8*, 39. https://doi.org/10.3390/informatics8020039

Issue Category	Definition	Examples	N (%)
10. Forgot to wear	Participants reported difficulties remembering to wear the device.	"I would forget to put it on because I don't like wearing a watch" and "I didn't like that sometimes it's difficult for me to remember to put it back on."	6 (4.8%)
11. Preference for certain features and reminders to be removed	Participants reported not liking certain device features like the built-in reminders.	"I don't like that it sometimes said 'feed me'…" and "I did not like the buzzing."	6 (4.8%)
12. Preference for additional features to be added	Participants reported wanting additional device features that were not currently there.	"It was helpful to have it, but I wish it would tell me how many calories I would burn with my activity" and "…I didn't like that you couldn't add certain activities like Zumba; they only had the pre-set exercises on the Fitbit."	5 (4.0%)
13. Other	Participants reported other psychological barriers that were not able to be categorized.	"I wasn't too into using the device" and "My attitude toward it was the only thing I didn't like" and "I sometimes would get anxiety around not meeting my activity goal" and "I do not think wearing a Bluetooth-activated gadget is healthy for me…"	4 (3.2%)
14. Lack of smartphone	Participants reported not owning a smartphone. (While it is not necessary for a Fitbit user to have a smartphone, it facilitates downloading the Fitbit app, accessing additional features, and syncing the data.)	"I don't have a smartphone to see my information."	3 (2.4%)
15. Misplaced device or charger	Participants reported misplacing their device or charger.	"I lost my charger and then the replacement got stolen or lost in the mail" and "I misplaced the Fitbit…"	2 (1.6%)
16. Broken device screen	Participants reported that the device screen was easily damaged.	"I liked everything except that it scratches easily."	2 (1.6%)
17. Trouble downloading Fitbit application	Participants reported problems downloading the Fitbit application onto their smartphone.	"I had to change phones and my new phone has less memory, so I couldn't download the app."	1 (0.8%)
**Total Count**			126

Orstad, S.L.; Gerchow, L.; Patel, N.R.; Reddy, M.; Hernandez, C.; Wilson, D.K.; Jay, M. Defining Valid Activity Monitor Data: A Multimethod Analysis of Weight-Loss Intervention Participants' Barriers to Wear and First 100 Days of Physical Activity. *Informatics* **2021**, *8*, 39.
https://doi.org/10.3390/informatics8020039
